# Hospital Reform Association

**Published:** 1898-02-26

**Authors:** 


### HOSPITAL REFORM ASSOCIATION.

Dr. Garrett Horder, honorary secretary Hospital Reform Association, writes enclosing a copy of the recommendations of the committee to which the scheme of reform laid before the Association had been referred. The recommendations are as follows :—

1. That in the casualty department of our general hospitals only cases of urgent importance should be attended to.

2. That in the out-patient department patients bringing notes from medical men should have, *cæteris paribus*, a prior claim to treatment. That a resident physician should be appointed whose duty it shall be to see all out-patients in the first instance and select those that require immediate treatment, and also decide which do not stand in need of hospital treatment. That after patients have received "first aid" their circumstances shall be inquired into by a competent officer. That the honorary medical officers shall not be required to treat more than twenty new cases at one sitting.

3 That the circumstances of all in-patients, with the exception of cases of accidents, should be carefully inquired into before admission.

4. That in the case of well-to-do people who are admitted to hospitals in consequence of accidents, &c., the hospitals should have the power at their discretion of recovering adequate fees for attendance.

5. In the case of smaller general hospitals, that the plan adopted at Oldham and Dorchester be recommended for trial.

6. That both in the large general hospitals and in the smaller ones out-patients coming from a distance should be requested to bring notes from medical men before being treated.

7. In the case of special hospitals—(*a*) that payment by patients should cease; (*b*) that the eligibility for free treatment should largely depend on the recommendation of private practitioners; (*c*) that some provision should be made outside the hospitals for people who are in a position to pay a reduced fee, but are not in a position to pay the ordinary fee of specialists.

